# Nasal carriage of *Staphylococcus aureus* in farm animals and breeders in north of Morocco

**DOI:** 10.1186/s12879-020-05329-4

**Published:** 2020-08-14

**Authors:** Nadira Mourabit, Abdelhay Arakrak, Mohammed Bakkali, Zeineb Zian, Joaira Bakkach, Amin Laglaoui

**Affiliations:** 1 Higher Institute of Nursing Professions and Technical Health of Tangier, Tetouan, Morocco; 2 Biotechnology and Biomolecule Engineering Research Laboratory, Faculty of Sciences and Techniques of Tangier, Abdelmalek Essaadi University, Tetouan, Morocco; 3 Biomedical Genomics and Oncogenetics Research Laboratory, Faculty of Sciences and Techniques of Tangier, Abdelmalek Essaadi University, Tetouan, Morocco

**Keywords:** *Staphylococcus aureus*, Nasal carriage, Animals, Breeders, MRSA, PVL, TSST-1, Morocco

## Abstract

**Background:**

The objectives of this study were to determine for the first time, in Morocco, the nasal carriage rate, antimicrobial susceptibility profiles and virulence genes of *Staphylococcus. aureus* isolated from animals and breeders in close contact.

**Methods:**

From 2015 to 2016, 421 nasal swab samples were collected from 26 different livestock areas in Tangier. Antimicrobial susceptibility phenotypes were determined by disk diffusion according to EUCAST 2015. The presence of *nuc*, *mec*A, *mec*C, lukS/F-PV, and *tst* genes were determined by Polymerase Chain Reaction (PCR) for all isolates.

**Results:**

The overall *S. aureus* nasal carriage rate was low in animals (9.97%) and high in breeders (60%) with a statistically significant difference, (OR = 13.536; 95% CI = 7.070–25.912; *p* < 0.001). In general, *S. aureus* strains were susceptible to the majority of antibiotics and the highest resistance rates were found against tetracycline (16.7% in animals and 10% in breeders). No Methicillin-Resistant *S. aureus* (MRSA) was detected in animals and breeders. A high rate of *tst* and lukS/F-PV genes has been recovered only from animals (11.9 and 16.7%, respectively).

**Conclusion:**

Despite the lower rate of nasal carriage of *S. aureus* and the absence of MRSA strains in our study, *S. aureus* strains harbored a higher frequency of *tst* and lukS/F-PV virulence genes, which is associated to an increased risk of infection dissemination in humans. This highlights the need for further larger and multi-center studies to better define the transmission of the pathogenic *S. aureus* between livestock, environment, and humans.

## Background

*Staphylococcus aureus*, especially, methicillin-resistant *S. aureus* strains (MRSA) are considered as major pathogens that infect and/or colonize both humans and animals [1]. Providing a reservoir of the pathogen, nasal carriage has been shown to be a predisposing factor for infections [2]. The emergence of MRSA is linked to the acquisition of the *mec*A gene that encodes for a low-affinity Penicillin-Binding Protein (PBP) [3] or its homologue *mec*C [4]. MRSA appeared firstly in a hospital in the early 1960s [5] and since then, it has been established around the world as an endemic hospital pathogen (Hospital Acquired-MRSA: HA-MRSA), and as Community-Acquired MRSA (CA-MRSA) causing human infections in the community [6]. In addition to humans, MRSA colonization and infection have also been reported in a variety of animal species in many countries [7]. These strains known as Livestock associated MRSA (LA-MRSA) strains had a genetic origin which is different to that of human strains previously described, with isolates mainly belonging to clonal complex CC398 [8].

The emergence of LA-MRSA has also been increasingly associated with alarming rates of MRSA infection and colonization among humans in contact with livestock, suggesting an increased risk of zoonotic transmission [9, 10]. These pathogenic strains could be subsequently dispersed to the environment and to other species [7] through food chain [11] and direct contact [12–14]. Numerous studies have indicated that the increasing rate of the incidence of antimicrobial resistance in *S. aureus* in animals is a result of the misuse and unjustified utilization of antibiotics [11, 14, 15] in animal husbandry either, for therapeutic, preventive purposes or as growth promoters (GPs). The Moroccan authorities through the national food safety office [16] have launched a surveillance program for some antimicrobials used in animals since 2001. However, the overuse of antibiotics still represents a challenging issue and GPs such as tetracycline are still permitted with a veterinary prescription in Morocco [17].

In African countries, studies about nasal carriage of LA-MRSA seem to be scarce. The first report of ST398 in humans in Africa was published by Elhani et al. [18] who isolated one MRSA-ST398-t899 in the nasal sample of a farmer from Tunisia. In Morocco, Mourabit et al. [19] documented a 1.4% nasal carriage rate of MRSA in healthy humans. Interestingly, this work identified for the first time in Morocco one isolate characterized as MRSA-ST398-t011.

Moreover, two other studies performed in Tangier on milk and milk products [20] and on cockroaches and the house flies [21] revealed higher incidence of *S. aureus* and suggested the potential role for food and insects as source and/or reservoir of *S. aureus* infection.

In Tangier livestock, little is known about antibiotic-resistant *S. aureus* carriage among animals, particularly farm animals which are in close contact with humans. The objective of this work was to determine the rate of nasal carriage of *S. aureus*, and the antimicrobial resistance and virulence genes in healthy farm animals and their breeders in Tangier.

## Methods

### Isolation and identification of nasal *S. aureus* isolates

**Fig. 1:**
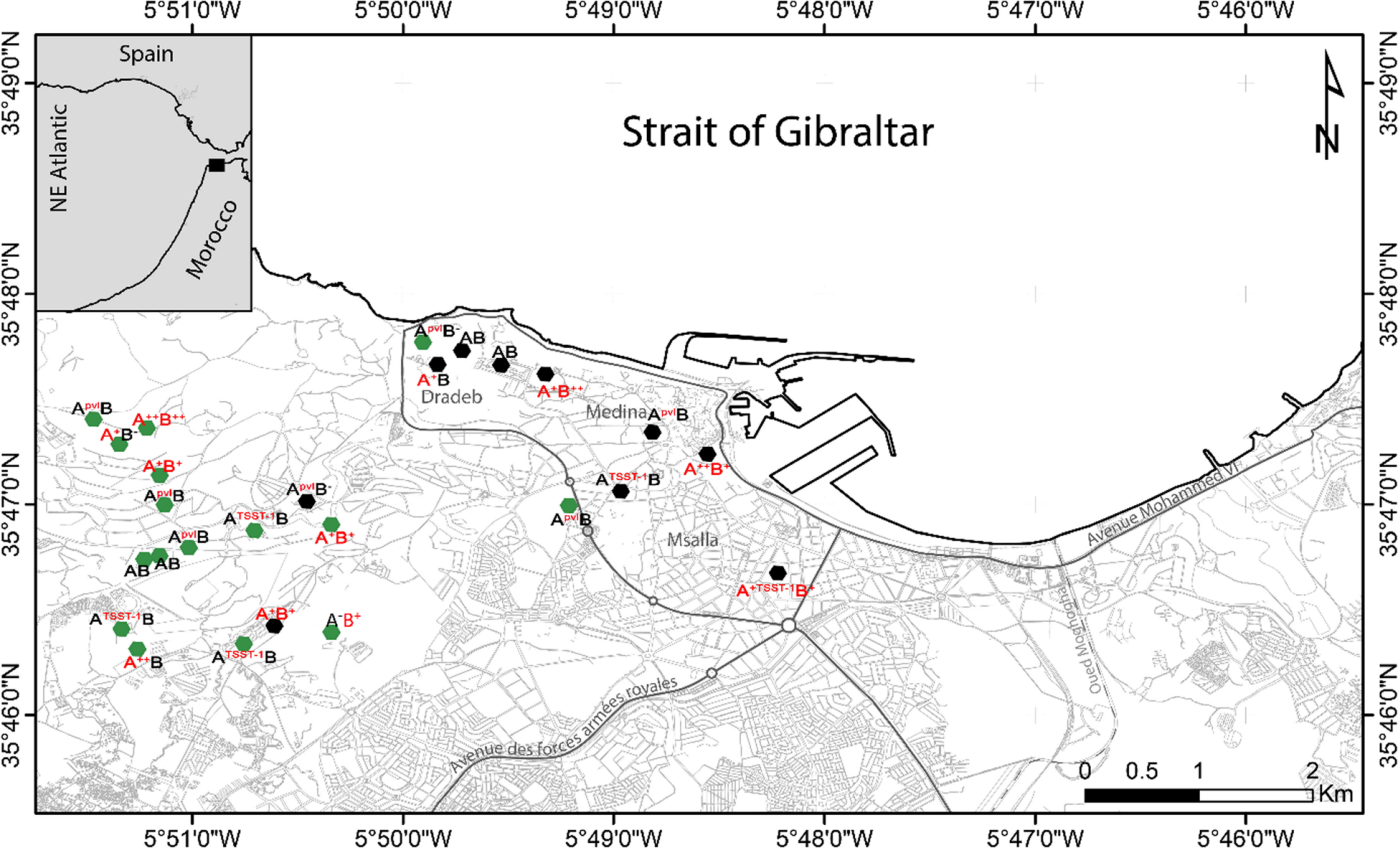
Geographical distribution of the 26 Livestock sampling areas in different regions of Tangier. Figure 1 made using the q geographic information system (QGIS) version 2.2 and GNU Image Manipulation Program (GIMP) version 2.10.20 software. Abbreviations: A^−^: Animal with no cases of *S. aureus*; A: Animal carrying multi-susceptible *S. aureus*; A^+^: Animal carrying *S. aureus* resistant to one antibiotic; A^++^: Animal carrying *S. aureus* resistant to two antibiotics; B^−^: Breeder with no cases of *S. aureus*; B: Breeder carrying multi-susceptible *S. aureus*; B^+^: Breeder carrying *S. aureus* resistant to one antibiotic; B^++^: Breeder carrying *S. aureus* resistant to two antibiotics; A/B^PVL^: Animal/Breeder carrying Panton–Valentine leukocidin-positive (PVL) *S. aureus*; A/B^TSST-1^: Animal/Breeder carrying Toxic Shock Syndrome Toxin (TSST-1)-positive *S. aureus*. The map was created by the authors using the q geographic information system (QGIS) version 2.2 and GNU Image Manipulation Program (GIMP) version 2.10.20 software

Livestock sampling areas included 16 small farms and 10 local sheep and goat farms in different regions of Tangier (Fig. 1). In general, the preference for selection of sampling sites was given to small farms or locals near the most populated areas of the city. In farms, the herd was composed of 20 to 50 heads. Among these, there were 4 to 15 cattle. The locals hosted less animals with a number not exceeding 20 heads and were composed essentially by sheep and goats (Table S1). This study was carried out between 2015 and 2016 and focused on clinically healthy animals and volunteers in close contact with these animals. To avoid a selection of resistant forms that can occur, no antibiotics within the last 3 months had been taken by animals [22]. Likewise, all the volunteers had no ongoing illness during the sampling period, and had not used antibiotics nor been hospitalized in the last three months [22].

A nasal swab of both nostrils was done simultaneously for each of the animals and volunteer breeders.

Consent and ethics approval: As the majority of breeders were illiterate, informed oral consent was obtained from all participants following the explanation of the study objectives. This was approved by an internal Ethics Committee in the Faculty of Sciences and Techniques of Tangier. Confidentiality of the participants was maintained using unique code.

### Phenotypic and molecular identification

To increase the chances of isolating the strains, the nasal samples were cultured in BHI medium and then incubated at 37 °C for 24 h. The isolation was carried out by successive subcultures on Chapman medium (Biorad) and on chromogenic media (UriSelect™ 4 dehydrated; Biorad), then incubated for 24 to 48 h at 37 °C.

Suspicious colonies were identified as *S. aureus* by colony morphology, Gram staining, catalase and DNase tests. One presumptively *S. aureus* isolate/sample was further confirmed by PCR for 16*S r*RNA and *nuc* genes, as previously described [3]. Susceptibility testing was performed with the disk diffusion method according to the recommendations of the European Committee on Antimicrobial Susceptibility Testing (EUCAST 2015) [23] for cefoxitin (30 μg), erythromycin (5 μg), lincomycin (15 μg), pristinamycin (15 μg), ciprofloxacin (5 μg), gentamicin (10 μg), tobramycin (10 μg), kanamycin (30 UI), tetracycline (30 UI), rifampicin (30 μg), chloramphenicol (10 μg), cotrimoxazole (23.75 ± 1.25 μg) and fusidic acid (10 μg). Inducible or constitutive lincomycin resistance was determined by the double disk diffusion test (D-test).

To avoid underestimating the prevalence of methicillin resistance, the presence of the *mec*A and *mec*C genes was determined by PCR in all these isolates, as previously described [3, 4]. All of the *S. aureus* isolates have been tested for the presence of lukS/F-PV and *tst* genes by PCR [24]. *S. aureus* strains ATCC 43300 (*mec*A-positive), ATCC 2011S359 (*mec*C-positive), ATCC 29213 (*nuc*-positive), MW2 (lukS/F-PV -positive) and FRI913 (*tst*-positive) were used as positive controls for PCRs.

All phenotypic and molecular tests were carried out in the Biotechnology and Biomolecule Engineering Research Laboratory.

### Statistical analyses

Comparisons between proportions were drawn with Fisher’s exact test and the Chi-squared test. Odds ratio (OR) and 95% confidence intervals (CI) were also calculated. Differences showing a *p* value < 0.05 were considered significant. Calculations were performed using SPSS version 20.0 software.

## Results

### Prevalence of *S. aureus* nasal carriage in farm animals

From 26 sampling sites (16 small farms and 10 local sheep and goat farms) in different regions of Tangier (Fig. 1; Table S1), a total of 421 different animals (cattle, sheep and goats) and 50 breeders were nasally screened. Phenotypic and molecular methods were able to isolate and identify forty-two (42/421, 9.97%) and thirty (30/50, 60%) strains from animals and breeders, respectively (OR = 13.536; 95% CI = 7.070–25.912; *p* < 0.001).

### Sensitivity to antibiotics

Twenty-two (73.3%) in breeders and thirty-two (76.2%) in animals of *S. aureus* had no resistance to the antibiotics tested. All the isolates were susceptible to cefoxitin, ciprofloxacin, trimethoprim/ sulfamethoxazole, tobramycin, gentamicin and chloramphenicol. The highest resistance rates were found for tetracycline (16.7 and 10%) and erythromycin (11.9 and 10%) in animals and breeders, respectively (Table 1). Screening for *mec*A and the *mec*C genes by PCR were negative. Of the resistant *S. aureus* isolates, six (6/8, 75%) and seven (7/10, 70%) recovered respectively from humans and animals presented resistance to one single antimicrobial drug. Co-resistance has been shown in two (2/8, 25%) isolates recovered from breeders and had as patterns (Tetracycline, Fusidic-acid); (Erythromycin, Fusidic-acid); and in three (3/10, 30%) isolates from animals with a unique pattern (Tetracycline, Erythromycin).

**Table 1:** Comparison of antimicrobial susceptibility profiles of isolates

	Animals *n* = 42 (%)	Breeders *n* = 30 (%)	*p*-value	OR (95% CI)
kanamycin	0 (0)	1 (3.3)	0.41	NA
Tobramycin	0 (0)	0 (0)	NA	NA
Gentamicin	0 (0)	0 (0)	NA	NA
Erythromycin	5 (11.9)	3 (10)	0.55	0.822 (0.181–3.740)
Ciprofloxacin	0 (0)	0 (0)	NA	NA
Tetracycline	7 (16.7)	3 (10)	0.32	0.556 (0.131–2.351)
Cotrimoxazole	0 (0)	0 (0)	NA	NA
Chloranphenicol	0 (0)	0 (0)	NA	NA
Fusidic-acid	1 (2.4)	3 (10)	0.19	0.566 (0.450–46.112)

*OR* odds ratio, *CI* confidence interval, *NA* not applicable (cannot be calculated due to zero cell)

### Exotoxin search

Of the 42 *S. aureus* isolates recovered from farm animals, 12 (28.57%) were toxinogenic. The genes encoding Panton Valentin Leucocidin toxin (PVL) and Toxic Shock Syndrome Toxin (TSST-1) were only identified in seven (16.7%) and five (11.9%) strains from animals. Among these strains, no one co-harbored lukS/F-PV and *tst* genes. Interestingly, no toxinogenic strain was recovered from breeders. All the toxinogenic *S. aureus* recovered from animals presented susceptibility to all tested antibiotics, except one isolate methicillin-susceptible *S. aureus* (MSSA)-TSST+, which showed resistance to tetracyclin.

## Discussion

In Morocco, livestock represents a large share of the Gross Domestic Product, which is between 25 and 30% according to the Ministry of Agriculture, Fisheries, Rural Development, Water and Forests [25]. This activity, which still plays an important socio-economic role, involves nearly 70% of the rural population [26]. However, animal farming also develops around urban centers where several farms with less than 20 to 60 heads or more are located in working-class areas or on the outskirts of the city (Fig. 1).

In Tangier, no data about LA-MRSA nasal carriage has been reported yet. Our results revealed statistically lower prevalence of *S. aureus* nasal carriage in farm animals compared to breeders with a statistically significant difference (OR = 13.536; 95% CI = 7.070–25.912; *p* < 0.001). Unfortunately, we could not compare our results to Moroccan studies due to the lack of such data at the national and local scale. In a report from Tunisia including 261 healthy animals, a relatively lower rate of *S. aureus* (6.5%) was reported with different rates depending on animal species. Similarly to our findings, this group showed that all *S. aureus* isolates were MSSA [26]. In contrast, some studies investigating the distribution of *S. aureus* in different ecological niches from livestock in Algeria [27, 28] reported prevalence rates that are ranging from 18 to 53%f or *S. aureus* and 5.4 to 7.6% for MRSA. Moreover, a recent study conducted by Dweba et al. [13], in South African livestock production systems identified MRSA isolates in 27% with a significant relationship (*p* < 0.001) with the animal host.

The current report revealed that all strains were MSSA and no significant differences were identified in antimicrobial resistance between LA-strains and human isolates (*p* > 0.05) (Table 1). These similar antimicrobial susceptibility profiles may be due to the common shared environment and/or with the urban lifestyle associated breeding. Hence, bacteria could spread from the community, environment to animals, or vice versa [12, 14, 20, 29]. The animals studied in our report were housed in, confined spaces located in working-class areas and fed mainly food waste or, on the outskirts of the city where sanitation infrastructure was lacking. Certainly, contamination by antibiotic residues contaminating food waste or waste water may be evoked [29], but this remains to be confirmed by other samples from the environment. In addition, transmission of livestock bacteria could also occurred through manure and/or when being carried in the air or in the dust, as it has been previously shown [12]. Moreover, breeders or persons in contact could also be contaminated when handling fields using manure from conventional farms as fertilizer [30].

On the other hand, transmission of resistant strains could also occur via insects as it has been suggested by Bouammama et al. [21] in a study conducted on cockroaches and the houseflies collected from residential areas in Tangier. *S. aureus* isolated from the external bodies of these two species of insects represented 6.7% (17 cases) of which, one was identified as MRSA.

As discussed above, in addition to animal-to-human, environment to human and or/animals, bacterial strains could also be transmitted via contaminated food such as milk and milk products or meat and meat products [14], when handling raw food with bare hands. In the same region of North of Morocco, another study conducted by Bendahou et al. [20] about milk and milk products revealed a higher rate of *S. aureus* (40%). The antimicrobial susceptibility-profile of the isolated staphylococcal strains in this study was similar to our findings (25 and 10% for tetracyclin and erythromycin resistance, respectively).

In Morocco, recent reports have documented the massive use of antibiotics in food producing animals for curative and prophylactic purposes, which may induce the development of resistance to antibiotics and persistence of residues in animal products [17]. Rahmatallah et al. reported that resistance to tetracycline exceeded 90% in the tested strains. This molecule which, is frequently sold as GPs has probably an important role in the selection of resistant strains [15, 17].

To the best of our knowledge, the present study reports for the first time toxinogenic *S. aureus* strains, recovered from healthy farm animals in Tangier. The detection of MSSA PVL+ and MSSA TSST-1+ is relevant due to the important role that these toxins seem to play in serious infections in humans and animals [28, 31]. Such pathogenic strains could spread between different ecological niches and through the food chain causing subsequently major problems for the healthcare system [27, 31]. Mairi et al. suggested that MSSA strains might become a permanent reservoir of the luk S/F-PV genes that caused human infections [27].

We have previously described nasal carriage of toxinogenic strains *S. aureus* in healthy persons, in the same geographical area with 11.5% (46/400) harboring luk S/F-PV and 15.5% (62/400) *tst* genes. Some of these TSST-1 strains were found to be MRSA; however, all of the PVL+ were MSSA [19].

The current study shows several limitations. The nasal carriage of *S. aureus* could be transient and therefore some cases may go unnoticed at the time of our investigation. Furthermore, other unexplored sites including the extra nasal ones could also host this germ. Additionally, our screening survey was limited to *tst* and lukS/F-PV genes. However, nasal carriage strains could harbor additional virulence factors that may aggravate their pathogenicity in both animals and humans. The limited size of the sample is another limitation of our work, which probably influenced the statistical results. Finally, the clonality of strains was not determined to check the type of ST found in animals and humans.

## Conclusion

Our nasal carriage study determined, for the first time in Tangier, a low prevalence of *S. aureus* with the absence of MRSA among farm animals. *S. aureus* strains harbored a higher rate of *tst* and lukS/F-PV genes, which is associated with an increased risk of dissemination of these strains in humans, animals and environment. Further studies are needed to better define the transmission of the pathogenic *S. aureus* between livestock, environment and humans.

## Supplementary information

**Additional file 1: Table S1**: Numbers, antimicrobial susceptibility and toxin gene profiles of the *Staphylococcus aureus* strains isolated from breeders and animals in Tangier.

## Data Availability

All data used to support the findings of this study are available from the corresponding author upon request.

## Abbreviations

*S. aureus*: *Staphylococcus aureus*
PCR: Polymerase Chain Reaction
MRSA: Methicillin-Resistant *S. aureus*
MSSA: Methicillin-susceptible *S. aureus*
PBP: Penicillin-Binding Protein
HA-MRSA: Hospital Acquired-MRSA
CA-MRSA: Community-Acquired MRSA
LA-MRSA: Livestock associated MRSA
CC: Clonal complex
GPs: Growth promoters
EUCAST: European Committee on Antimicrobial Susceptibility Testing
OR: Odds ratio
CI: Confidence intervals
PVL: Panton Valentin Leucocidin toxin
TSST-1: Toxic Shock Syndrome Toxin

## References

[1] Nemeghaire S, Argudin MA, Haesebrouck F, Butaye P (2014). Epidemiology and molecular characterization of methicillin-resistant *Staphylococcus aureus* nasal carriage isolates from bovines. BMC Vet Res.

[2] Wertheim HF, Melles DC, Vos MC, Van Leeuwen W, Van Belkum A, Verbrugh HA (2005). The role of nasal carriage in *Staphylococcus aureus* infections. Lancet Infec Dis.

[3] Maes N, Magdalena J, Rottiers S, De Gheldre Y, Struelens MJ (2002). Evaluation of a triplex polymerase chain reaction (PCR) assay to discriminate *Staphylococcus aureus* from coagulase-negative staphylococci (CoNS) and determine methicillin resistance from blood cultures. J Clin Microbiol.

[4] Garcia-Alvarez L, Holden MT, Lindsay H, Webb CR, Brown DF, Curran MD (2011). Methicillin-resistant *Staphylococcus aureus* with a novel *mec*A homologue in human and bovine populations in the UK and Denmark: a descriptive study. Lancet Infect Dis.

[5] Jevons MP, Coe AW, Parker MT (1963). Methicillin resistance in staphylococci. Lancet..

[6] Vandenesch F, Naimi T, Enright MC, Lina G, Nimmo GR, Heffeman H (2003). Community acquired methicillin-resistant *Staphylococcus aureus* carrying Panton-Valentine leukocidin genes: worldwide emergence. Emerg Infect Dis.

[7] Cuny C, Friedrich A, Kozytska S, Layer F, Nubel U, Ohlsen K (2010). Emergence of methicillin-resistant *Staphylococcus aureus* (MRSA) in different animal species. Int J Med Microbiol.

[8] Guardabassi L, Stegger M, Skov R (2007). Retrospective detection of methicillin resistant and susceptible *Staphylococcus aureus* ST398 in Danish slaughter pigs. Vet Microbiol.

[9] Hanselman BA, Kruth SA, Rousseau J, Low DE, Willey BM, McGeer A (2006). Methicillin resistant *Staphylococcus aureus* colonization in veterinary personnel. Emerg. Infect Dis.

[10] Spoor LE, McAdam PR, Weinert LA, Rambaut A, Hasman H, Aarestrup FM (2013). Livestock origin for a human pandemic clone of community-associated methicillin-resistant *Staphylococcus aureus*. mBio.

[11] Kluytmans JA (2010). Methicillin-resistant *Staphylococcus aureus* in food products: cause for concern or case for complacency?. Clin Microbiol. Infect.

[12] Schulz J, Friese A, Klees S, Tenhagen BA, Fetsch A, Rosler U (2012). Longitudinal study of the contamination of air and of soil surfaces in the vicinity of pig barns by livestock-associated methicillin resistant *Staphylococcus aureus*. Appl Environ Microbiol.

[13] Dweba CC, Zishiri O, El Zowalaty M (2018). Methicillin-resistant *Staphylococcus aureus*: livestock-associated, antimicrobial, and heavy metal resistance. Infect Drug Resist.

[14] Gundogan N, Citak S, Yucel N, Devren A (2005). A note on the incidence and antibiotic resistance of *Staphylococcus aureus* isolated from meat and chicken samples. Meat Sci.

[15] Aarestrup FM (1999). Association between consumption of antimicrobial agents in animal husbandry and the occurrence of resistant bacteria among food animals. Int J Antimicrob Agents.

[16] Office national de sécurité sanitaire des aliments (ONSSA). Requirements for poultry production. 2015. Available from http://www.onssa.gov.ma/fr/index.php?option=com_content&view=article&id=187&Itemid=126. (Accessed 10 March 2015).

[17] Rahmatallah N, El Rhaffouli H, Lahlou Amine I, Sekhsokh Y, FassiFihri O, El Houadfi M (2018). Consumption of antibacterial molecules in broiler production in Morocco. Vet Med Sci.

[18] Elhani D, Gharsa H, Kalai D, Lozano C, Gomez P, Boutheima J (2015). Clonal lineages detected among tetracycline resistant MRSA isolates of a Tunisian hospital, with detection of lineage ST398. J Med Microbiol.

[19] Mourabit N, Arakrak A, Bakkali M, Laglaoui A (2017). Nasal carriage of sequence type 22 MRSA and livestock-associated ST398 clones in Tangier, Morocco. J Infect Dev Ctries.

[20] Bendahou A, Abid M, Bouteldoun N, Catelejine D, Lebbadi M (2009). Staphylocoque à coagulase positive entérotoxinogène dans le lait et les produits laitiers, lben et jben, dans le nord du Maroc. J Infect DevCtries.

[21] Bouamamaa L, Sorlozano A, Laglaoui A, Lebbadi M, Aarab A, Gutierrez J (2010). Profils de résistance aux antibiotiques des souches bactériennes isolées de Periplaneta americana et Muscadomestica à Tanger, Maroc. J Infect DevCtries.

[22] Levy SB (2002). Factors impacting on the problem of antibiotic resistance. J Antimicrob Chemotherapy.

[23] The European Committee on Antimicrobial Susceptibility Testing. Breakpoint tables for interpretation of MICs and zone diameters. Version 5.0. 2015. https://www.eucast.org/fileadmin/src/media/PDFs/EUCAST_files/Breakpoint_tables/v_5.0_Breakpoint_Table_01.pdf (Accessed on 10 April 2015).

[24] Denis O, Deplano A, De Beehouwer H, Hallin M, Huysmans G, Garrino MG (2005). Polyclonal emergence and importation of community-acquired methicillin-resistant *Staphylococcus aureus* strains harbouring Panton-valentine leucocidin genes in Belgium. J Antimicrob Chemother.

[25] Ministry of Agriculture, Fisheries, Rural Development, Water and Forests (MAF). 2014. http://www.agriculture.gov.ma/en.

[26] Gharsa H, Ben Slama K, Gomez-Sanz E, Lozano C, Zarazaga M, Messadi L (2015). Molecular characterization of *Staphylococcus aureus* from nasal samples of healthy farm animals and pets in Tunisia. Vector Borne Zoonotic Dis.

[27] Mairi A, Touati A, Pantel A, Zenati K, Martinez AY, Dunyach-Remy C (2019). Distribution of Toxinogenic methicillin-resistant and methicillin-susceptible *Staphylococcus aureus* from different ecological niches in Algeria. Toxins (Basel).

[28] Agabou A, Ouchenane Z, NgbaEssebe C, Khemissi S, Chehboub MTE, Chehboub IB (2017). Emergence of nasal carriage of ST80 and ST152 PVL + *Staphylococcus aureus* isolates from livestock in Algeria. Toxins (Basel).

[29] Ezzariai A, Hafidi M, Khadra A, Aemig Q, El Fels L, Barret M (2018). Human and veterinary antibiotics during composting of sludge or manure: global perspectives on persistence, degradation, and resistance genes. J Hazard Mater.

[30] Casey JA, Curriero FC, Cosgrove SE, Nachman KE, Schwartz BS (2013). High-density livestock operations, crop field application of manure, and risk of community-associated methicillin-resistant *Staphylococcus aureus* infection in Pennsylvania. JAMA Intern Med.

[31] Otto M (2014). *Staphylococcus aureu*s toxins. Curr Opin Microbiol.

