# Supplementary material for: Nasal carriage of Staphylococcus aureus in farm animals and breeders in north of Morocco
Source: BMC Infect Dis. 2020 Aug 14;20:602. doi: 10.1186/s12879-020-05329-4 (PMC7429463; doi:10.1186/s12879-020-05329-4)
Supplement: Supplementary file 1 — Additional file 1: Table S1: Numbers, antimicrobial susceptibility and toxin gene profiles of the Staphylococcus aureus strains isolated from breeders and animals in Tangier. [file 12879_2020_5329_MOESM1_ESM.docx]

Table S1: Numbers, antimicrobial susceptibility and toxin gene profiles of the *Staphylococcus aureus* strains isolated from breeders and animals in Tangier

| Animals  (n/n’)  Breeders  (n/n’) | | Ferbruary | March | April | May | June | July | August | September | October | November | December | January | Ferbruary | *S. aureus* | Cefoxitin | Kanamycin | Tobramycin | Gentamycin | Erythromycin | Lyncomycin | Ciprofloxacin | Tetracyclin | Cotrimoxasol | Chloranphenicol | Fusidic-acid | PVL | TSST-1, |
| --- | --- | --- | --- | --- | --- | --- | --- | --- | --- | --- | --- | --- | --- | --- | --- | --- | --- | --- | --- | --- | --- | --- | --- | --- | --- | --- | --- | --- |
| Farm 1 | Animals **(0/27)** | **ŠŠ** |  |  |  |  |  |  |  |  |  |  |  |  | **-** | NA | NA | NA | NA | NA | NA | NA | NA | NA | NA | NA | NA | NA |
|  | Breeders **(1/2)** | **ŠŠ** |  |  |  |  |  |  |  |  |  |  |  |  | **+** | **S** | **R** | **S** | **S** | **S** | **S** | **S** | **S** | **S** | **S** | **S** | **-** | **-** |
| Local 1 | Animals **(2/14)** | **ŠŠ** |  |  |  |  |  |  |  |  |  |  |  |  | **+** | **S** | **S** | **S** | **S** | **S** | **S** | **S** | **S** | **S** | **S** | **R** | **-** | **-** |
|  |  |  |  |  |  |  |  |  |  |  |  |  |  |  | **+** | **S** | **S** | **S** | **S** | **S** | **S** | **S** | **S** | **S** | **S** | **S** | **-** | **-** |
|  | Breeders **(1/1)** | **ŠŠ** |  |  |  |  |  |  |  |  |  |  |  |  | **+** | **S** | **S** | **S** | **S** | **S** | **S** | **S** | **S** | **S** | **S** | **R** | **-** | **-** |
| Farm 2 | Animals **(2/18)** |  | **ŠŠ** |  |  |  |  |  |  |  |  |  |  |  | **+**  **+** | **S**  **S** | **S**  **S** | **S**  **S** | **S**  **S** | **S**  **S** | **S**  **S** | **S**  **S** | **S**  **S** | **S**  **S** | **S**  **S** | **S**  **S** | **-** | + |
|  | Breeders **(2/2)** |  | **ŠŠ** |  |  |  |  |  |  |  |  |  |  |  | **+**  **+** | **S** | **S** | **S** | **S** | **S** | **S** | **S** | **S** | **S** | **S** | **S** | **-** | **-** |
| Local 2 | Animals (**1/8)** |  | **ŠŠ** |  |  |  |  |  |  |  |  |  |  |  | **+** | **S** | **S** | **S** | **S** | **S** | **S** | **S** | **S** | **S** | **S** | **S** | + | **-** |
|  | Breeders **(0/1)** |  | **ŠŠ** |  |  |  |  |  |  |  |  |  |  |  | **-** | NA | NA | NA | NA | NA | NA | NA | NA | NA | NA | NA | NA | NA |
| Farm 3 | Animals **(1/24**) |  |  | **ŠŠ** |  |  |  |  |  |  |  |  |  |  | **+** | **S** | **S** | **S** | **S** | **R** | **S** | **S** | **R** | **S** | **S** | **S** | **-** | **-** |
|  | Breeders **(1/2)** |  |  | **ŠŠ** |  |  |  |  |  |  |  |  |  |  | **+** | **S** | **S** | **S** | **S** | **S** | **S** | **S** | **S** | **S** | **S** | **S** | **-** | **-** |
| Farm 4 | Animals **(1/18)** |  |  | **ŠŠ** |  |  |  |  |  |  |  |  |  |  | **+** | **S** | **S** | **S** | **S** | **S** | **S** | **S** | **S** | **S** | **S** | **S** | **-** | + |
|  | Breeders **(1/2)** |  |  | **ŠŠ** |  |  |  |  |  |  |  |  |  |  | **+** | **S** | **S** | **S** | **S** | **S** | **S** | **S** | **S** | **S** | **S** | **S** | **-** | **-** |
| Local 3 | Animals (**2/12**) |  |  |  | **ŠŠ** |  |  |  |  |  |  |  |  |  | **+** | **S** | **S** | **S** | **S** | **S** | **S** | **S** | **R** | **S** | **S** | **S** | **-** | + |
|  |  |  |  |  |  |  |  |  |  |  |  |  |  |  | **+** | **S** | **S** | **S** | **S** | **S** | **S** | **S** | **S** | **S** | **S** | **S** |  |  |
|  | Breeders **(1/1)** |  |  |  | **ŠŠ** |  |  |  |  |  |  |  |  |  | **+** | **S** | **S** | **S** | **S** | **S** | **S** | **S** | **R** | **S** | **S** | **S** | **-** | **-** |
| Farm 5 | Animals **(1/19)** |  |  |  | **ŠŠ** |  |  |  |  |  |  |  |  |  | **+** | **S** | **S** | **S** | **S** | **S** | **S** | **S** | **S** | **S** | **S** | **S** | + | **-** |
|  | Breeders **(1/3)** |  |  |  | **ŠŠ** |  |  |  |  |  |  |  |  |  | **+** | **S** | **S** | **S** | **S** | **S** | **S** | **S** | **S** | **S** | **S** | **S** | **-** | **-** |
| Farm 6 | Animals (**2/17)** |  |  |  | **ŠŠ** |  |  |  |  |  |  |  |  |  | **+** | **S** | **S** | **S** | **S** | **S** | **S** | **S** | **S** | **S** | **S** | **S** | **-** | **-** |
|  |  |  |  |  |  |  |  |  |  |  |  |  |  |  | **+** | **S** | **S** | **S** | **S** | **S** | **S** | **S** | **S** | **S** | **S** | **S** | **-** | **-** |
|  | Breeders **(1/2)** |  |  |  | **ŠŠ** |  |  |  |  |  |  |  |  |  | **+** | **S** | **S** | **S** | **S** | **S** | **S** | **S** | **S** | **S** | **S** | **S** | **-** | **-** |
| Local 4 | Animals **(1/14)** |  |  |  |  | **ŠŠ** |  |  |  |  |  |  |  |  | **+** | **S** | **S** | **S** | **S** | **S** | **S** | **S** | **R** | **S** | **S** | **S** | **-** | **-** |
|  | Breeders **(1/2)** |  |  |  |  | **ŠŠ** |  |  |  |  |  |  |  |  | **+** | **S** | **S** | **S** | **S** | **S** | **S** | **S** | **S** | **S** | **S** | **S** | **-** | **-** |
| Farm 7 | Animals **(3/27)** |  |  |  |  | **ŠŠ** |  |  |  |  |  |  |  |  | **+** | **S** | **S** | **S** | **S** | **R** | **S** | **S** | **S** | **S** | **S** | **S** | **-** | **-** |
|  |  |  |  |  |  |  |  |  |  |  |  |  |  |  | **+** | **S** | **S** | **S** | **S** | **S** | **S** | **S** | **S** | **S** | **S** | **S** | **-** | **-** |
|  |  |  |  |  |  |  |  |  |  |  |  |  |  |  | **+** | **S** | **S** | **S** | **S** | **S** | **S** | **S** | **S** | **S** | **S** | **S** | **-** | **-** |
|  | Breeders **(1/3)** |  |  |  |  | **ŠŠ** |  |  |  |  |  |  |  |  | **+** | **S** | **S** | **S** | **S** | **R** | **S** | **S** | **S** | **S** | **S** | **S** | **-** | **-** |
| Local 5 | Animals **(2/8)** |  |  |  |  |  | **ŠŠ** |  |  |  |  |  |  |  | **+**  **+** | **S**  **S** | **S**  **S** | **S**  **S** | **S**  **S** | **S**  **S** | **S**  **S** | **S**  **S** | **S**  **S** | **S**  **S** | **S**  **S** | **S**  **S** | **-**  **-** | +  - |
|  | Breeders **(1/1)** |  |  |  |  |  | **ŠŠ** |  |  |  |  |  |  |  | **+** | **S** | **S** | **S** | **S** | **S** | **S** | **S** | **S** | **S** | **S** | **S** | **-** | **-** |
| Farm 8 | Animals **(1/23)** |  |  |  |  |  | **ŠŠ** |  |  |  |  |  |  |  | **+** | **S** | **S** | **S** | **S** | **S** | **S** | **S** | **S** | **S** | **S** | **S** | **-** | **-** |
|  | Breeders **(2/3)** |  |  |  |  |  | **ŠŠ** |  |  |  |  |  |  |  | **+** | **S** | **S** | **S** | **S** | **S** | **S** | **S** | **S** | **S** | **S** | **S** | **-** | **-** |
|  |  |  |  |  |  |  |  |  |  |  |  |  |  |  | **+** | **S** | **S** | **S** | **S** | **S** | **S** | **S** | **S** | **S** | **S** | **S** | **-** | **-** |
| Local 6 | Animals **(2/12)** |  |  |  |  |  |  | **ŠŠ** |  |  |  |  |  |  | **+** | **S** | **S** | **S** | **S** | **R** | **S** | **S** | **R** | **S** | **S** | **S** | **-** | **-** |
|  |  |  |  |  |  |  |  |  |  |  |  |  |  |  | **+** | **S** | **S** | **S** | **S** | **S** | **S** | **S** | **S** | **S** | **S** | **S** | **-** | **-** |
|  | Breeders **(2/2)** |  |  |  |  |  |  | **ŠŠ** |  |  |  |  |  |  | **+** | **S** | **S** | **S** | **S** | **R** | **S** | **S** | **S** | **S** | **S** | **S** | **-** | **-** |
|  |  |  |  |  |  |  |  |  |  |  |  |  |  |  | **+** | **S** | **S** | **S** | **S** | **S** | **S** | **S** | **S** | **S** | **S** | **S** | **-** | **-** |
| Farm 9 | Animals **(2/17)** |  |  |  |  |  |  | **ŠŠ** |  |  |  |  |  |  | **+** | **S** | **S** | **S** | **S** | **S** | **S** | **S** | **S** | **S** | **S** | **S** | + | **-** |
|  |  |  |  |  |  |  |  |  |  |  |  |  |  |  | **+** | **S** | **S** | **S** | **S** | **S** | **S** | **S** | **S** | **S** | **S** | **S** | **-** | **-** |
|  | Breeders **(1/1)** |  |  |  |  |  |  | **ŠŠ** |  |  |  |  |  |  | **+** | **S** | **S** | **S** | **S** | **S** | **S** | **S** | **S** | **S** | **S** | **S** | **-** | **-** |
| Farm 10 | Animals **(1/11)** |  |  |  |  |  |  |  | **ŠŠ** |  |  |  |  |  | **+** | **S** | **S** | **S** | **S** | **S** | **S** | **S** | **S** | **S** | **S** | **S** | **-** | + |
|  | Breeders **(1/2)** |  |  |  |  |  |  |  | **ŠŠ** |  |  |  |  |  | **+** | **S** | **S** | **S** | **S** | **S** | **S** | **S** | **S** | **S** | **S** | **S** | **-** | **-** |
| Local 7 | Animals **(4/14)** |  |  |  |  |  |  |  |  | **ŠŠ** |  |  |  |  | **+** | **S** | **S** | **S** | **S** | **S** | **S** | **S** | **R** | **S** | **S** | **S** | **-** | **-** |
|  |  |  |  |  |  |  |  |  |  |  |  |  |  |  | **+** | **S** | **S** | **S** | **S** | **S** | **S** | **S** | **S** | **S** | **S** | **S** | **-** | **-** |
|  |  |  |  |  |  |  |  |  |  |  |  |  |  |  | **+** | **S** | **S** | **S** | **S** | **S** | **S** | **S** | **S** | **S** | **S** | **S** | **-** | **-** |
|  |  |  |  |  |  |  |  |  |  |  |  |  |  |  | **+** | **S** | **S** | **S** | **S** | **S** | **S** | **S** | **S** | **S** | **S** | **S** | **-** | **-** |
|  | Breeders **(1/1)** |  |  |  |  |  |  |  |  | **ŠŠ** |  |  |  |  | **+** | **S** | **S** | **S** | **S** | **S** | **S** | **S** | **R** | **S** | **S** | **R** | **-** | **-** |
| Farm 11 | Animals **(2/19)** |  |  |  |  |  |  |  |  | **ŠŠ** |  |  |  |  | **+** | **S** | **S** | **S** | **S** | **S** | **S** | **S** | **S** | **S** | **S** | **S** | + | **-** |
|  |  |  |  |  |  |  |  |  |  |  |  |  |  |  | **+** | **S** | **S** | **S** | **S** | **S** | **S** | **S** | **S** | **S** | **S** | **S** | **-** | **-** |
|  | Breeders **(1/1)** |  |  |  |  |  |  |  |  | **ŠŠ** |  |  |  |  | **+** | **S** | **S** | **S** | **S** | **S** | **S** | **S** | **S** | **S** | **S** | **S** | **-** | **-** |
| Local 8 | Animals **(1/12)** |  |  |  |  |  |  |  |  |  | **ŠŠ** |  |  |  | **+** | **S** | **S** | **S** | **S** | **S** | **S** | **S** | **S** | **S** | **S** | **S** | + | **-** |
|  | Breeders **(1/1)** |  |  |  |  |  |  |  |  |  | **ŠŠ** |  |  |  | **+** | **S** | **S** | **S** | **S** | **S** | **S** | **S** | **S** | **S** | **S** | **S** | **-** | **-** |
| Farm 12 | Animals **(1/18)** |  |  |  |  |  |  |  |  |  | **ŠŠ** |  |  |  | **+** | **S** | **S** | **S** | **S** | **S** | **S** | **S** | **S** | **S** | **S** | **S** | + | **-** |
|  | Breeders **(1/3)** |  |  |  |  |  |  |  |  |  | **ŠŠ** |  |  |  | **+** | **S** | **S** | **S** | **S** | **S** | **S** | **S** | **S** | **S** | **S** | **S** | **-** | **-** |
| Local 9 | Animals (3/10) |  |  |  |  |  |  |  |  |  |  | **ŠŠ** |  |  | **+** | **S** | **S** | **S** | **S** | **S** | **S** | **S** | **S** | **S** | **S** | **S** | **-** | **-** |
|  |  |  |  |  |  |  |  |  |  |  |  |  |  |  | **+** | **S** | **S** | **S** | **S** | **S** | **S** | **S** | **S** | **S** | **S** | **S** | **-** | **-** |
|  |  |  |  |  |  |  |  |  |  |  |  |  |  |  | **+** | **S** | **S** | **S** | **S** | **S** | **S** | **S** | **S** | **S** | **S** | **S** | **-** | **-** |
|  | Breeders **(2/2)** |  |  |  |  |  |  |  |  |  |  | **ŠŠ** |  |  | **+** | **S** | **S** | **S** | **S** | **S** | **S** | **S** | **S** | **S** | **S** | **S** | **-** | **-** |
|  |  |  |  |  |  |  |  |  |  |  |  |  |  |  | **+** | **S** | **S** | **S** | **S** | **S** | **S** | **S** | **S** | **S** | **S** | **S** | **-** | **-** |
| Farm 13 | Animals (**1/24**) |  |  |  |  |  |  |  |  |  |  | **ŠŠ** |  |  | **+** | **S** | **S** | **S** | **S** | **R** | **S** | **S** | **R** | **S** | **S** | **S** | **-** | **-** |
|  | Breeders **(1/2)** |  |  |  |  |  |  |  |  |  |  | **ŠŠ** |  |  | **+** | **S** | **S** | **S** | **S** | **R** | **S** | **S** | **S** | **S** | **S** | **R** | **-** | **-** |
| Local 10 | Animals **(2/9)** |  |  |  |  |  |  |  |  |  |  |  | **ŠŠ** |  | **+** | **S** | **S** | **S** | **S** | **S** | **S** | **S** | **S** | **S** | **S** | **S** | **-** | **-** |
|  |  |  |  |  |  |  |  |  |  |  |  |  |  |  | **+** | **S** | **S** | **S** | **S** | **S** | **S** | **S** | **S** | **S** | **S** | **S** | **-** | **-** |
|  | Breeders **(3/3)** |  |  |  |  |  |  |  |  |  |  |  | **ŠŠ** |  | **+** | **S** | **S** | **S** | **S** | **S** | **S** | **S** | **S** | **S** | **S** | **S** | **-** | **-** |
|  |  |  |  |  |  |  |  |  |  |  |  |  |  |  | **+** | **S** | **S** | **S** | **S** | **S** | **S** | **S** | **S** | **S** | **S** | **S** | **-** | **-** |
|  |  |  |  |  |  |  |  |  |  |  |  |  |  |  | **+** | **S** | **S** | **S** | **S** | **S** | **S** | **S** | **S** | **S** | **S** | **S** | **-** | **-** |
| Farm 14 | Animals **(1/13)** |  |  |  |  |  |  |  |  |  |  |  | **ŠŠ** |  | **+** | **S** | **S** | **S** | **S** | **S** | **S** | **S** | **S** | **S** | **S** | **S** | + | **-** |
|  | Breeders **(0/3)** |  |  |  |  |  |  |  |  |  |  |  | **ŠŠ** |  | **-** | NA | NA | NA | NA | NA | NA | NA | NA | NA | NA | NA | NA | NA |
| Farm 15 | Animals **(2/12)** |  |  |  |  |  |  |  |  |  |  |  |  | **ŠŠ** | **+** | **S** | **S** | **S** | **S** | **R** | **S** | **S** | **S** | **S** | **S** | **S** | **-** | **-** |
|  |  |  |  |  |  |  |  |  |  |  |  |  |  |  | **+** | **S** | **S** | **S** | **S** | **S** | **S** | **S** | **S** | **S** | **S** | **S** | **-** | **-** |
|  | Breeders **(1/2)** |  |  |  |  |  |  |  |  |  |  |  |  | **ŠŠ** | **-** | NA | NA | NA | NA | NA | NA | NA | NA | NA | NA | NA | NA | NA |
| Farm 16 | Animals **(1/21)** |  |  |  |  |  |  |  |  |  |  |  |  | **ŠŠ** | **+** | **S** | **S** | **S** | **S** | **S** | **S** | **S** | **R** | **S** | **S** | **S** | **-** | **-** |
|  |  |  |  |  |  |  |  |  |  |  |  |  |  |  | **+** | **S** | **S** | **S** | **S** | **S** | **S** | **S** | **S** | **S** | **S** | **S** | **-** | **-** |
|  | Breeders **(1/3)** |  |  |  |  |  |  |  |  |  |  |  |  | **ŠŠ** | **+** | **S** | **S** | **S** | **S** | **S** | **S** | **S** | **R** | **S** | **S** | **S** | **-** | **-** |
|  |  |  |  |  |  |  |  |  |  |  |  |  |  |  | **+** | **S** | **S** | **S** | **S** | **S** | **S** | **S** | **S** | **S** | **S** | **S** | **-** | **-** |
| Total | animal **42/421** |  |  |  |  |  |  |  |  |  |  |  |  |  |  |  |  |  |  |  |  |  |  |  |  |  |  |  |
|  | breeders **30/50** |  |  |  |  |  |  |  |  |  |  |  |  |  |  |  |  |  |  |  |  |  |  |  |  |  |  |  |

Farm: The herd of goats, cattle and sheep was between 20 to 50 heads. Among them, there were 4 to 15 cattle; Local: The herd of goats and sheep was between 8 à 20 heads;

Abbreviations:

n: number of *S. aureus* strains isolated; n’=number of samples; NA: Not applicable; PVL: Panton–Valentine leukocidin-positive; R: Resistant; S: Susceptible; *S. aureus: Staphylococcus aureus;* **ŠŠ**: Sampling areas; TSST-1: Toxic shock syndrome toxin-1.
